# Larval habitat characterization of *Anopheles darlingi* from its northernmost geographical distribution in Chiapas, Mexico

**DOI:** 10.1186/s12936-015-1037-0

**Published:** 2015-12-22

**Authors:** Cuauhtémoc Villarreal-Treviño, R. Patricia Penilla-Navarro, M. Guadalupe Vázquez-Martínez, David A. Moo-Llanes, Jana C. Ríos-Delgado, Ildefonso Fernández-Salas, Américo D. Rodríguez

**Affiliations:** Centro Regional de Investigación en Salud Pública/Instituto Nacional de Salud Pública (CRISP/INSP), 19 Poniente y 4ta Avenida Norte, C.P. 30700 Tapachula, Chiapas Mexico

**Keywords:** *Anopheles darlingi*, Lacandon forest, Malaria, Anopheline habitats

## Abstract

**Background:**

*Anopheles darlingi* is considered the most efficient malaria vector in the Neotropical region. In Mexico, its role as an incriminated vector of *Plasmodium* has not been confirmed in the Lacandon forest. Similarly, knowledge about bionomic and larval ecology is scarce. The study aim was to identify and describe the larval habitats of *An. darlingi* in Chiapas, México.

**Methods:**

Standard larval collections were performed in the Lacandon forest region and in the Soconusco region of southern Chiapas from January 2010 to April 2014, including dry and rainy seasons. Mean larval density of *An. darlingi* was estimated according to hydrological types, and associations between the presence of *An*. *darlingi* and environmental factors including ecological parameters and geographic positions were statistically analysed.

**Results:**

One hundred and twelve aquatic habitats were analysed, 80 from the Lacandon forest region and 32 from the Soconusco region; 94.64 % of these sites presented anopheline larvae. In total, 10,977 larvae belonging to 11 *Anopheles* species were collected. The 19 (out of 112) larval habitats positive to *An. darlingi* were: rain puddles (26.32 %), ground pools (21.05 %), ponds (15.79 %), ditches (15.79 %), river margins (10.53 %) and streams (10.53 %). Overall, the average (±SD) larval density was 6.60 ± 2.41 larvae per dip. Multiple logistic regression analysis showed that temporary habitats, green algae presence and stagnant water were associated with *An. darlingi* larval presence. The positive habitats were found in the Lacandon forest region during the rainy season (May–September). No specimens were found in the Soconusco region of the coastal plain of Chiapas.

**Conclusion:**

The mosquito *An. darlingi* larval habitats were found in different hydrological types. The habitat stability, presence of algae and water current were the main factors for *An. darlingi* larval occurrence. The information on the characteristics of the larval habitats of *An. darlingi* will be useful in sustainable programmes for malaria control in the Lacandon forest region, Chiapas.

**Electronic supplementary material:**

The online version of this article (doi:10.1186/s12936-015-1037-0) contains supplementary material, which is available to authorized users.

## Background

Malaria continues to be a global public health problem, and it is considered the most important vector-borne infectious disease in the world, including Mexico [1]. *Anophelesdarlingi*, a member of the Albitarsis section of the Nyssorhynchus subgenus is considered the dominant and most dangerous malaria vector in the Neotropical region [2, 3]. It is widely distributed in Central and South America, from southern Mexico to northern Argentina. It has not been described in Nicaragua and Costa Rica [2, 4, 5], and it was recently reported in Panama [6].

In Iquitos, Peru, *An. darlingi* populations increased dramatically in the 1990s, after deforestation and the creation of new human settlements and roads [7]. The increase in malaria cases also occurred specifically due by *Plasmodium falciparum* [8]. In Central and South America, this species is considered the primary malaria vector of *Plasmodium vivax* and *P. falciparum* [9, 10]. In susceptibility experiments with *P. falciparum* in Belize, it was found that *An. darlingi* is the most susceptible species, with 41.0 % positivity in their salivary glands and more than 200 sporozoites per gland [11]. In Mexico, it has not been found infected in its natural state. However, in the Lacandon forest region, this species is considered to participate as a secondary vector transmitting malaria [12, 13].

In Belize, larvae prefer those habitats with available organic matter found in river margins, and shaded areas with submerged plants in fluvial environments [14]; preference is mainly associated with floating detritus [15]. In Brazil and Colombia, larvae are usually found in shaded breeding habitats with clean running water and organic matter [16, 17]. This species is commonly found in rural areas and small localities, but it is unlikely to be found in undisturbed areas [18].

Between 2006 and 2008, the state of Chiapas presented the highest number of malaria cases in Mexico (58.3 % of all cases in the country), mainly along the Mexico-Guatemala border (Lacandon forest and Soconusco focus, Chiapas) [19]. In Mexico, *An. darlingi* was reported in the Anaite village alongside the Usumacinta River in the Lacandon forest (Chiapas), and in Teapa (Tabasco) at 72 m above sea level (masl) [20, 21]. Larval habitats were described as sunny, clean fresh water with low movement, pH of 7.1–7.2, temperature between 17.5 and 33 °C, and always present in low densities [22]. However, this information is outdated and the larval ecology studies for this species have not been continued, in spite that the Lacandon forest region is hyperendemic for malaria. During the 1960s and 1970s, native Mexicans from the Yucatan Peninsula and later from other northern states of Mexico migrated to Chiapas specifically to the Lacandon forest region [23]. Furthermore, this area has been subject to extensive and accelerated deforestation from 1954 to 2000, with almost 30 % of trees felled. The new settlements, as well as unregulated logging and forest fires caused changes in the orography of the region. However, the impact of these changes in vectors population density and transmission dynamics are still unknown. Similar changes are known to result in the occurrence of new larval habitats and the increase of adult *An. darlingi* densities in the Amazon rain forest of Peru [7, 24].

This study aims to identify and describe *An. darlingi* larval habitats in two geographic regions of southern Chiapas, Mexico. These findings will be useful in determining the current biology and ecology of the species, and will help in planning prevention and control activities for this important malaria vector in the Lacandon forest region.

## Methods

### Study design

Larvae collection sites were determined according to previous reports for *An. darlingi* distribution [21, 22] and potential sites by a prediction model in the Malaria Atlas Project [25] in the Lacandon forest region (17°37′30.69″N, 92°01′41.56″W to 16°5′8.91″N, 90°28′0.37″W) and the southern coastal plain named the Soconusco region (15°39′21.05″N, 93°22′24.95″W to 14°32′6.50″N, 92°13′23.33″W). Both areas are endemic for malaria and are used as migration routes by people traveling to northern Mexico and the United States [26]. The Lacandon forest region, including “The Montes Azules Biosphere Reserve”, is delimited by the Lacantun, Jatate and Lacanja rivers, all of which flow into the Usumacinta River. The climate in the region is tropical wet (Koppen classification, *Af*, *Am*), with abundant rain from May to November. Annual median temperature is 24–26 °C, and the annual precipitation ranges between 2500 and 3500 mm [27]. Altitude for the region is 120–840 masl [23]. The Soconusco region has a tropical wet and dry climate (Koppen classification, Aw), with a rainy season from May to October, annual mean temperature of 27 °C and annual precipitation of 2800 mm. The altitude in the region ranges from sea level next to the Pacific Ocean to 500 masl at the foothills [27]. The Chiapas coastal plain comprises extensive banana and mango plantations, swamps, and a livestock grazing areas (0–150 masl); while the regions near the mountains comprise large coffee plantations (150–550 masl).

### *Anopheles darlingi* field collection

Diverse larval habitats were visited from January 2010 to April 2014, including dry and rainy seasons. In the Lacandon forest, larvae were collected from rural and semi-urban areas, alongside highways and roads. In the Soconusco region, located at the south of the coastal plain of Chiapas, different agro-ecological zones were visited starting on the border with Guatemala to the northwest 150 km along the coastline, and from sea level to 500 masl, following the course of the Coatán River.

Larval index (LI) or larval density was expressed as total number of larvae per dip/the number of dips × the number of habitats [28]. Sampled water was poured into a white tray, using a standard 500 ml dipper [29]. Larvae (I to IV instar) were counted and packed in plastic bags (Whirl–Pak^®^) and placed into an ice chest. The anopheline larvae sampled from each larval habitat were reared in separate trays until adulthood in the insectary at the Centro Regional de Investigación en Salud Pública (CRISP) in Tapachula, Chiapas, to determine the species according to a morphological based key [30].

For each aquatic habitat, environmental variables were recorded during larval sampling, including hydrological types [31]. Average water depth was measured from three different points of each larval habitat using a 1 m wooden ruler introduced it until reached the solid bottom. Turbidity was determined visualizing a water sample in a transparent bottle and placing it on a light background and recorded as clear or turbid [32]. Vegetation coverage, surface detritus, presence of green algae, and intensity of light were estimated by visual assessment as the percentage of total surface covered. The aquatic vegetation was categorized as emergent, floating or submerged and none when were not present. Emergent plants include both aquatic and immersed terrestrial vegetation. Water current, was estimated as stagnant, slow or high [33, 34]. Habitat stability was categorized as permanent or temporal. Permanent habitats were considered as those that remained during the dry season and temporary as those that appears only during the rainy season. The elevation and coordinates (longitude and latitude) of all selected breeding sites were recorded in situ using a portable GPS receiver. The distribution map and the calculation of distances between the larval habitats and the nearest house were made using the software Esri^®^ArcMap the geomatics suite Arc GIS 10.3 software.

### Data analysis

Mann–Whitney U test was used to compare the larval index or density of anopheline species collected between Lacandon forest and Soconusco regions. Pearson correlation was used to determine the association between *An. darlingi* larval density with rainfall and the relationship between *An. darlingi* larvae presence with other anopheline species coexisting in the same larval habitat. One-way analysis of variance (ANOVA) was used to analyse the abundance between species. Multiple logistic regression analyses were used to determine the association between environmental variables and the occurrence of *An. darlingi* larvae. Statistical analysis was performed using JMP 7.0.1 software (SAS Institute, Cary, NC, USA).

## Results

### *Anopheles* species abundance

**Fig. 1:**
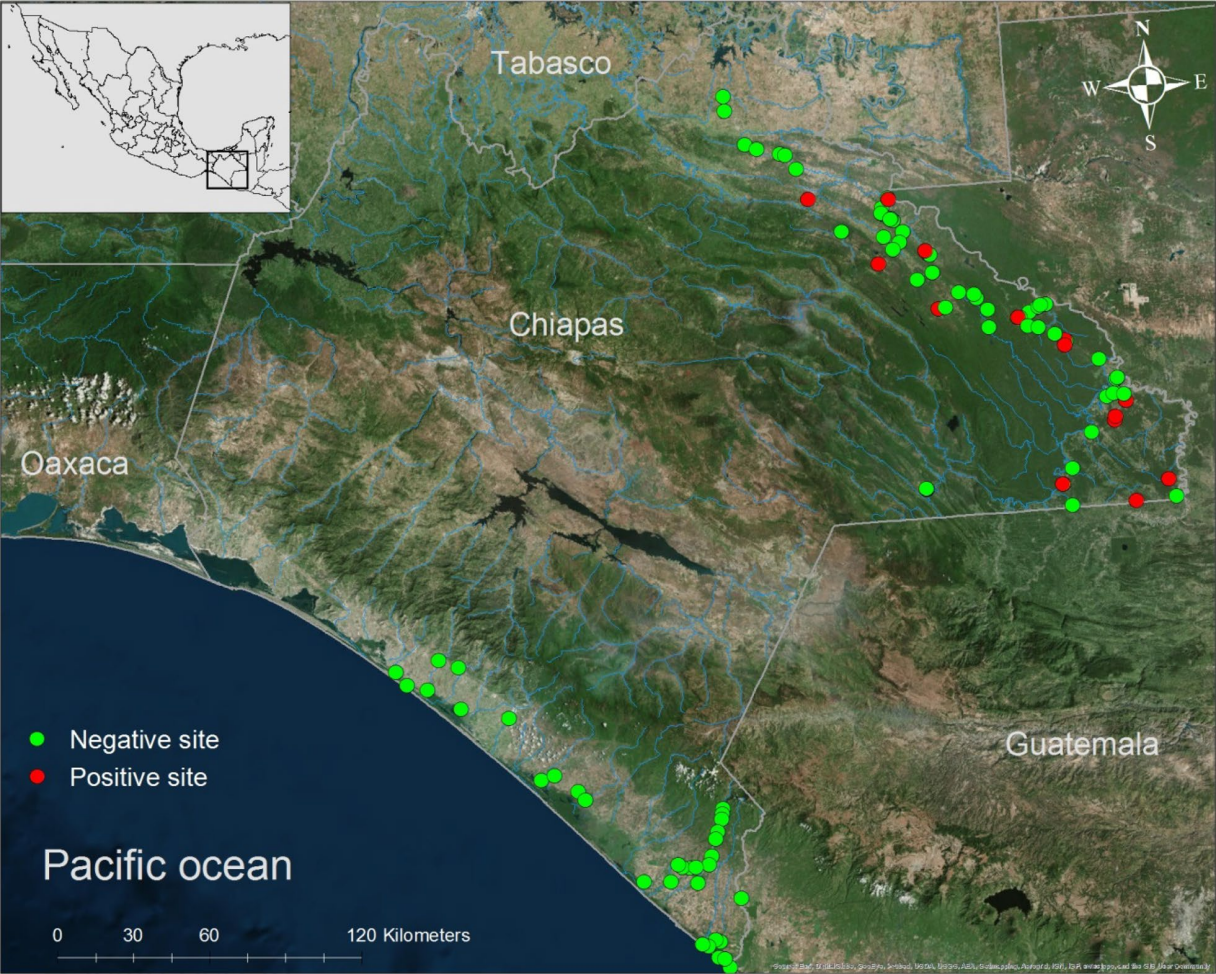
Study area indicating the negative and positive larval habitats of *Anopheles darlingi* in Chiapas, Mexico

**Fig. 2:**
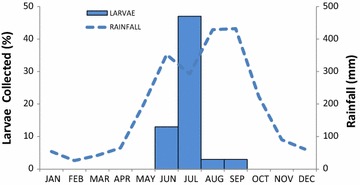
Monthly frequency of pooled *Anopheles darlingi* larval population sampling in the Lacandon forest region, Chiapas, Mexico 2010–2014

Each aquatic habitat was visited from 19 to 25 times during 4 years. A total of 10,977 anopheline larvae were collected of which 10,421 adults were obtained, belonging to 11 *Anopheles* species. Larval index (LI) showed significant differences between the Lacandon forest (LI = 2.9) and Soconusco regions (LI = 3.6) (Mann–Whitney U = 682; *p* < 0.0001). *An. darlingi* was found only in the Lacandon forest region, in northeastern Chiapas (between 16°9′36.00″N, 90°52′48.10″W; and 17°14′15.50″N, 91°44′28.99″W to Mexican border with Guatemala). No specimen was found in the south coastal region of Chiapas (Fig. 1). The *An. darlingi* larvae occurrence was positively correlated with the rainy season in the Lacandon forest region (R^2^ = 0.609; *p* = 0.036) (Fig. 2).

**Fig. 3:**
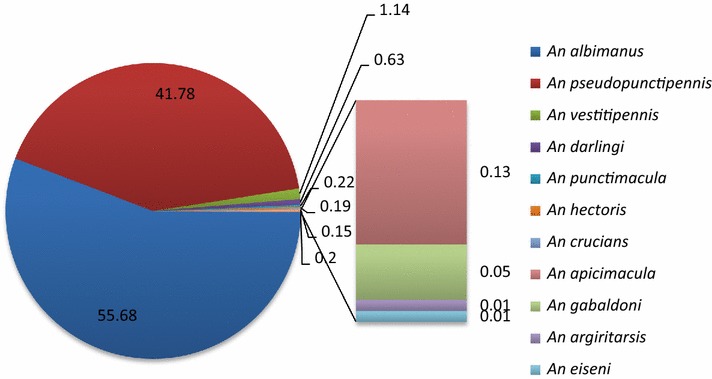
Anopheline species composition and abundance (percent) in the Lacandon forest and Soconusco regions, Chiapas, Mexico

Species abundance showed significant differences (P < 0.05): *Anopheles albimanus* (mean = 71.6; SD ± 128.97) (55.68 %) and *Anopheles pseudopunctipennis* (mean = 92.6 ± 154.17) (41.78 %), were the most abundant in comparison with *Anopheles vestitipennis* (mean = 11.9 ± 13.10) (1.14 %), *An. darlingi* (mean = 3.5 ± 2.412) (0.63 %), *Anopheles punctimacula* (mean = 4.6 ± 2.073) (0.22 %), *Anopheles hectoris* (mean = 20.0 ± 0.0) (0.19 %), *Anopheles crucians* (mean = 4.0 ± 4.24) (0.15 %), *Anopheles apicimacula* (mean = 4.7 ± 3.51) (0.13 %), *Anopheles gabaldoni* (mean = 2.5 ± 0.70) (0.05 %), *Anopheles argiritarsis* (mean = 1.0 ± 0.0) (0.01 %) and *Anopheles eiseni* (mean = 1.0 ± 0.0) (0.01 %) (F = 5.2; df = 10,120; *p* = 0.0001) (Fig. 3).

In the hydrological types positive for *An. darlingi* larvae, other species were found coexisting in the following total percentages: *An. pseudopunctipennis* (45.66 %), *An. albimanus* (42.72 %), *An. vestitipennis* (6.52 %), *An. darlingi* (4.73 %) and *An. crucians* (0.35 %). There was a positive correlation between presence of *An. darlingi* and *An. albimanus* (R^2^ = 0.957; *p* = 0.001), *An. pseudopunctipennis* (R^2^ = 0.777, *p* = 0.040) and *An. crucians* (R^2^ = 0.785, *p* = 0.036), but not with *An. vestitipennis* (R^2^ = 0.635, *p* = 0.125) (Table 1).

**Table 1:** Anopheline species composition (%) coexisting with *An. darling* larvae in the Lacandon forest, Chiapas

Hydrologic type	Total larvae	Ditches	Ground pools	Ponds	Rain puddles	River margins	Streams
*An. pseudopunctipennis*	637	1.72	5.33	87.44	2.82	1.72	0.94
*An. albimanus*	596	0	29.36	41.10	19.29	4.69	5.53
*An. vestitipennis*	91	76.92	0	0	23.07	0	0
*An. darlingi*	66	7.57	22.72	27.27	16.66	7.57	18.18
*An. crucians*	5	0	0	20	0	0	80
Total	1395	6.16	16.06	58.85	11.83	3.15	3.94

### Habitat types of *Anopheles darlingi*

A total of 112 aquatic habitats were sampled throughout the study, 80 for the Lacandon forest region and 32 for the Soconusco region. A total of 106 (94.64 %) larval habitats were positive for anopheline larvae and only 19 (16.94 %) were positive for *An. darlingi* larvae. All *An. darlingi* positive habitats were present in a deforested area of the Lacandon forest region.

A total of 66 *An. darlingi* larvae were captured during the study in six different hydrological types distributed as follows: ponds (27.27 %), ground pools (22.72 %), streams (18.18 %), rain puddles (16.66 %), ditches (7.57 %), and river margins (7.57 %) (Table 1). The mean (±SD) larval density in all positive hydrological types was low with a 6.6 ± 0.2.41 LI (Table 2). To observe the different hydrological types in more detail see (Additional file 1). There was no association between the hydrological type and *An. darlingi* larval abundance (Table 3).

**Table 2:** Larval abundance of *Anopheles darlingi* in different hydrological types in the Lacandon forest, Chiapas, Mexico

Hydrological types	No sampled	No positive	% positive	Larval density^a^
Rain puddles	24	5	26.32	2.20 ± 1.304
Ground pools	6	4	21.05	3.75 ± 1.500
Ponds	14	3	15.79	6.00 ± 3.000
Ditches	4	3	15.79	1.66 ± 1.155
River margins	17	2	10.53	2.50 ± 0.707
Streams	6	2	10.53	6.00 ± 4.243
River Pools	5	0	0	0
Lagoons	4	0	0	0
Total	80	19	100	6.60 ± 2.41

^a^Mean ± SD

**Table 3:** Logistic regression for *Anopheles darlingi* larvae abundance in the Lacandon forest and Soconusco regions, Chiapas, México

Variable	B	SE	df	Sig	OR	95 % CI for OR	
						Lower	Upper
Interception	−0.015	0.175		0.279	−0.087	−0.362	0.331
Water depth			2	0.110			
0–100	0.043	0.021	1	0.043	2.054	0.001	0.084
101–300	0.000	0.000	1	0.934	65,535.000	0.000	65,536.000
301–800	0.052	0.162	1	0.965	0.321	−0.269	0.373
Turbidity	−0.68	0.072	1	0.347	−0.944	−0.211	0.075
Coverage			2	0.200			
0–30 %	−0.090	0.107	1	0.401	−0.843	−0.303	0.122
31–60 %	0.000	0.000	1	0.690	65,535.000	26.000	65,546.000
61–100 %	−0.082	0.150	1	0.530	−0.548	−0.379	0.215
Detritus			2	0.600			
Low	−0.384	0.142	1	0.008	−2.699	−0.666	−0.102
Moderate	−0.080	0.115	1	0.486	−0.699	−0.308	0.147
High	0.000	0.000	1	0.230	65,535.000	19,849.000	65,595.000
Presence of algae	0.151	0.075	1	0.046	2.021	0.003	0.298
Intensity of light			2	0.079			
Light	0.000	0.000	1	0.580	65,535.000	18,679.000	65,538.000
Light/shade	0.054	0.129	1	0.960	0.418	−0.202	0.310
Shade	0.108	0.115	1	0.351	0.937	−0.120	0.336
Vegetation			2	0.550			
Emergent	0.236	0.138	1	0.123	1.704	−0.039	0.510
Floating/submerged	0.000	0.000	1	0.583	65,535.000	1657.000	65,536.000
None	0.216	0.094	1	0.890	2.304	0.030	0.403
Water current			2	0.000			
Stagnant	1.000	0.110	1	0.000	9.080	0.781	1.218
Slow	0.096	0.081	1	0.236	1.192	−0.064	0.256
High	0.000	0.000	1	0.260	65,535.000	1487.000	65,539.000
Habitat stability	0.188	0.081	1	0.022	2.327	0.028	0.349
Elevation	0.134	0.268	1	0.616	0.503	−0.396	0.665
Distance to nearest house			2	0.580			
0–2.66 km	0.085	0.140	1	0.545	0.607	−0.193	0.363
2.67–5.89 km	0.121	0.161	1	0.456	0.748	−0.199	0.441
5.90–12.1 km	0.000	0.000	1	0.630	65,535.000	5276.000	65,535.000
Region	0.085	0.058	1	0.650	1.468	−0.030	0.199
Hydrological type			10	0.810			
Ditches	0.000	0.011	1	0.976	−0.030	−0.022	0.022
Gravel pits	0.283	0.292	1	0.334	0.971	−0.296	0.863
Ground pools	0.193	0.284	1	0.498	0.680	−0.371	0.757
Irrigation canal	0.142	0.243	1	0.559	0.586	−0.339	0.624
Lagoon	0.135	0.277	1	0.627	0.488	−0.415	0.686
Marshes	0.159	0.241	1	0.505	0.669	−0.312	0.630
Ponds	0.349	0.241	1	0.150	1.452	−0.128	0.827
Rain puddles	0.140	0.318	1	0.660	0.441	−0.491	0.772
River margins	−0.047	0.303	1	0.877	−0.156	−0.649	0.554
River pools	0.140	0.337	1	0.678	0.417	−0.529	0.810
Pools	0.000	0.000	1	0.690	65,535.000	527.000	65,535.000
Streams	0.135	0.277	1	0.090	0.488	−0.415	0.686

*OR* odds ratio, *CI* confidence interval, *Sig* significant, *df* degrees of freedom

### Habitat description of *Anopheles darlingi*

Multiple logistic regression showed that presence of algae, habitat stability, and water current were the principal factors associated for *An. darlingi* larvae occurrence in the habitat (Table 3). Green algae were presented in 84.21 % of the larval habitats and significantly associated (*p* = 0.046) with the occurrence of larvae. The *An. darlingi* larvae were mainly present in temporary habitats during the rainy season (*p* = 0.022). Larval density was positively associated with water movement (*p* = 0.000), finding larvae in stagnant water or slow movement, but never in high movement (Table 3).

## Discussion

In the present study *An. darlingi* larvae were present in different hydrological types, associated with *An. albimanus, An. pseudopunctipennis* and *An. crucians*. These associations were consistent with the data documented from Belize, where *An. darlingi* was also found coexisting with *An. albimanus* and *An. pseudopunctipennis* [14]. *Anopheles darlingi* larvae were found only during the rainy season, from June to September, with a peak in July. During August and September it was very difficult to find *An. darlingi* larvae, because the water current at hydrological types such as streams, river margins, river pools, and ditches increased with the rain, washing away larval breeding habitats [35]. Also, hydrological types such as rain puddles, ground pools, and ponds increased in size during the rainy season, making it difficult to find *An. darlingi* larvae. About 53 % of the breeding habitats disappeared during the dry season, reducing the availability of larval habitats of *An. darlingi* [35].

The results in this study showed that rain puddles had the greatest percentage of positive habitats followed by ground pools and ponds; the less productive are the river margins and streams. These larval habitats in the Lacandon forest region were the consequence of product human activities in the area; including excessive logging, construction of rural and public roads, and the introduction of livestock. Similar results were found in the Peruvian tropical rainforest, where a strong association of large ponds and larvae was detected due to changes in the natural habitats caused by human alterations [7]. That promoted the development of *An. darlingi,* in such a way that biting rate was 278 times higher in deforested regions as compared to forested, and the presence of human populations was indicator of *An. darlingi* larvae [24]. Water bodies such as lakes, ponds and river margins, inside the “Montes Azules” ecological reserve, did not present *An. darlingi* larvae; this is consistent with the findings of studies done in the Brazilian Amazon, where the lowest anthrophilic mosquito index found was in the Maraca ecologic reserve [18]. In the Peruvian Amazon, *An. darlingi* larvae were breeding less frequently in water bodies that were surrounded predominantly by forest [24]. In Colombia, *An. darlingi* is a riverine mosquito that prefers living in rural areas of low elevation within the jungle [17]. Similarly in Panama, it prefers to inhabit areas that have been little disturbed by humans [6]. These differences in *An. darlingi* habitat preference of either forest areas or human-altered areas is very complex and will depend on the regional ecology of each site [36, 37].

Preferred breeding habitats for *An. darlingi* have been described in different environments of Central and South America [14–18, 35]. In Mexico, *An. darlingi* has been only reported in the Lacandon forest region in Chiapas and Teapa, Tabasco in the 1940s and 1950s [20–22]. These old reports describe larval habitats as: sunny, low-movement freshwater bodies in the river margins of the Usumacinta River. In this study, no *An. darlingi* larvae were found in the Usumacinta River margins. This is probably because it requires intensified sampling mainly in the dry season, because that is when ponds are formed in the margins of the river, so this species occurrence should not rule out in some areas of the Usumacinta River. Another probable habitat for *An. darlingi* in Mexico is the state of Quintana Roo, because it borders with Belize, which has an important role in malaria transmission. However, *An. darlingi* has not been found in this region to date [38]. This study was performed over 4 years because the larvae were found in very low densities and sampling was made difficult by the inaccessibility of the larval habitats. Similar reports such as one from Belize mention that *An. darlingi* breeding habitats are difficult to find, such that the authors did not collect any *An. darlingi* larvae after 2 years of intensive search. However, *An. darlingi* habitats were found in open water areas nearby rivers, with the help of satellite imagery [39].

The results of this study showed no difference between sunny and shaded habitats, indicating that the larvae do not have a defined preference for light. This contrasts with the findings of previous studies from Mexico [19, 20], where it is mentioned that the *An. darlingi* larvae prefer sunny habitats, just as in Peru, where *An. darlingi* larval densities increased with the amount of light [24]. Reports from Panama [6], Belize [14], and Colombia [17], mention that larvae prefer shaded habitats. Reports from Panamá and Belize state that *An darlingi* larvae prefer clear water with a slow flow, which is consistent with the results of this study [14, 17]. In this study, the presence of algae in the breeding habitats was a positive indicator for the presence of *An. darlingi* larvae as found in Belize and Peru studies [14, 24]. This positive correlation can be explained because larvae feed on the algae and use the algae to hide from predators [40]. Larval density in the Lacandon forest breeding habitats is in concordance with the densities reported in other countries, which are never abundant [5].

In Mexico, *An. darlingi* is not considered a primary malaria vector in the Lacandon forest region [12], for several possible reasons. One hypothesis is the low larval and therefore adult densities. In a study of incrimination of *Anopheles* spp in the Lacandon forest, only 21 (0.6 %) specimens of *An. darlingi* were collected with human baiting during 6 months (June–November) and all were negative for malaria parasites [12]. This shows the low density of this species in the Lacandon forest region. Another explanation could be that the population belongs to genotype 2 (northern population), which is reported in Belize, Guatemala, and Colombia, where this genotype is considered a secondary vector. Genotype 1 (southern population) is reported in the Amazon forest and Brazil, where it is abundant and is considered a primary vector in places with high incidence of *P. vivax* and *P. falciparum* malaria [41–43]. None of the reported molecular studies for this important malaria vector have included Mexican populations. Therefore, the genetic relationship between Mexican *An. darlingi* populations and those of Central and South America is unknown. It is necessary to continue studying this important malaria vector in the Neotropical region, with the aim to elucidate its role in the transmission of malaria in Mexico and take the necessary control measures.

*Anopheles darlingi* larvae were found only in the Lacandon forest region (Fig. 1). However, periodical sampling is required in the Soconusco region in southern Chiapas, as well as to extend the study area to other states such as Quintana Roo, Tabasco and Veracruz because the presence of this mosquito is expected at these places [44].

Taking into account this study findings and as part of a programme of vector larval control strategies, priority should be given to control positive types of habitat of *An darlingi* and coexisting species that occur at the start of the rainy season, especially in breeding sites containing algae with little current or stagnant water and located near to human settlements. To control these breeding site habitats an environmental management like removing algae and/or a biorational insecticide application can be undertaken. This study is the first of its kind by the amplitude and diversity of habitats that were examined in the Lacandon forest and Soconusco region and is useful to develop sustainable control strategies in this important focus of malaria in the South of Mexico.

## Conclusions

This study has demonstrated that the habitat stability, presence of algae and water current were the main factors for *An. darlingi* larval occurrence. The information on the characteristics of the larval habitats of *An. darlingi* will be useful in sustainable programmes for malaria control in the region of the Lacandon forest, Chiapas.

## Additional file

10.1186/s12936-015-1037-0 Hydrological types positive and negative to *Anopheles darlingi* larvae in the Lacandon forest region, Chiapas, México.

## Abbreviations

LI: larval index
SD: standard deviation
