# Supplementary material for: Larval habitat characterization of Anopheles darlingi from its northernmost geographical distribution in Chiapas, Mexico
Source: Malar J. 2015 Dec 22;14:517. doi: 10.1186/s12936-015-1037-0 (PMC4688970; doi:10.1186/s12936-015-1037-0)
Supplement: Supplementary file 1 — 10.1186/s12936-015-1037-0 Hydrological types positive and negative to Anopheles darlingi larvae in the Lacandon forest region, Chiapas, México. [file 12936_2015_1037_MOESM1_ESM.pptx]

## Slide 1
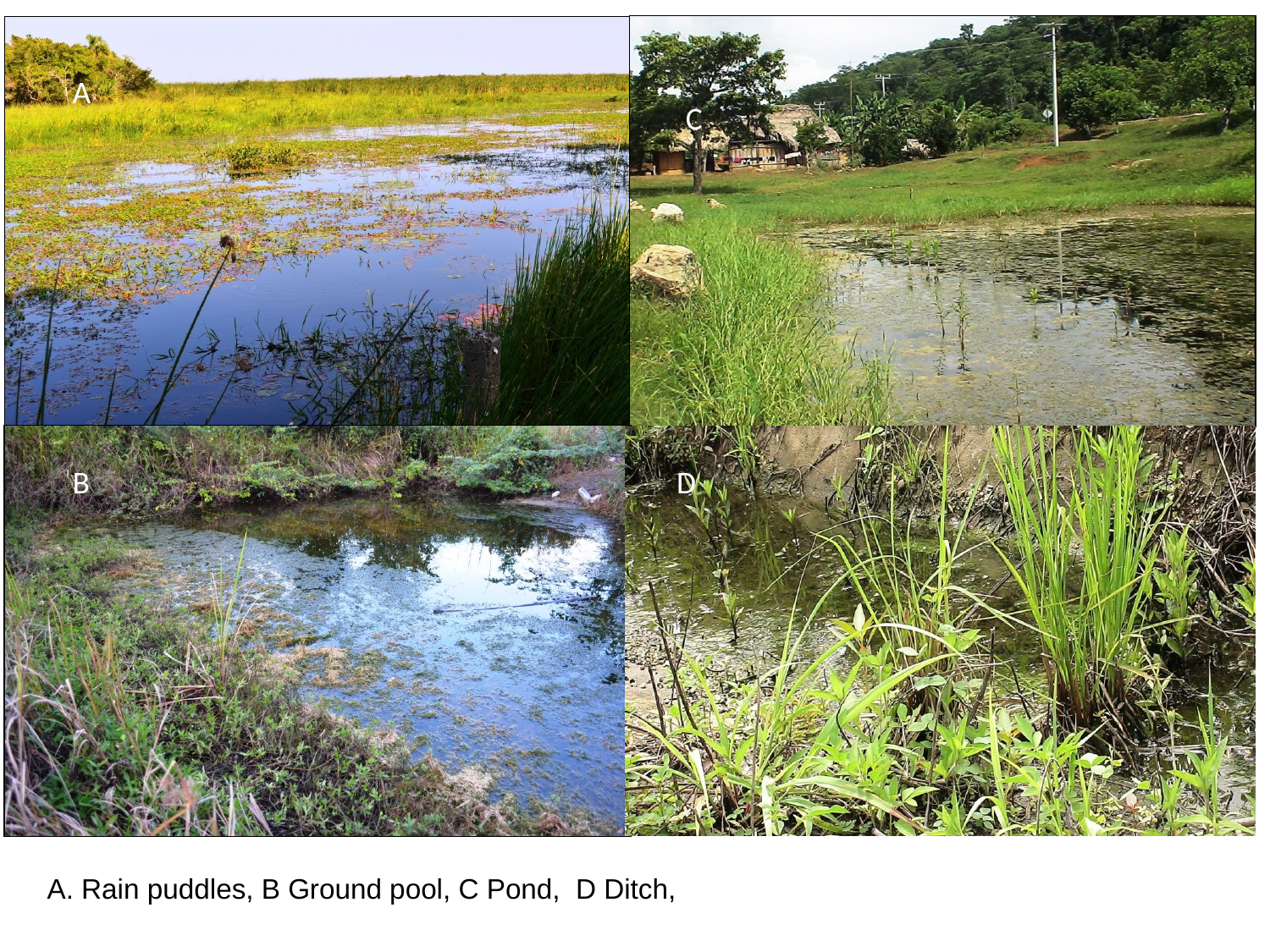

A
C
B
D
A. Rain puddles, B Ground pool, C Pond, D Ditch,

## Slide 2
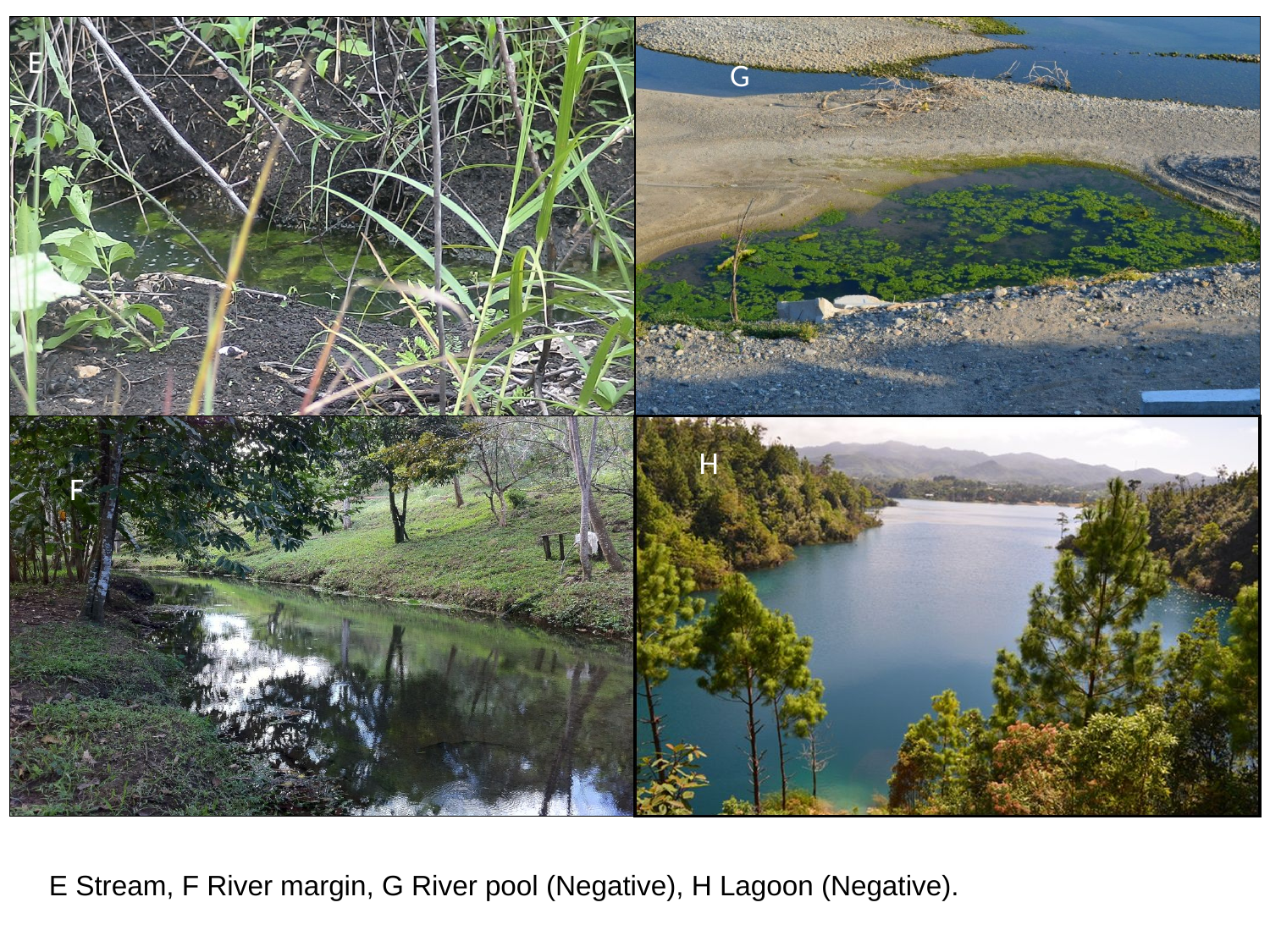

E
G
H
F
E Stream, F River margin, G River pool (Negative), H Lagoon (Negative).
